# Multi-omics network characterization reveals novel microRNA biomarkers and mechanisms for diagnosis and subtyping of kidney transplant rejection

**DOI:** 10.1186/s12967-021-03025-8

**Published:** 2021-08-13

**Authors:** Yuxin Lin, Liangliang Wang, Wenqing Ge, Yu Hui, Zheng Zhou, Linkun Hu, Hao Pan, Yuhua Huang, Bairong Shen

**Affiliations:** 1 Department of Urology, The First Affiliated Hospital of Soochow University, Suzhou, 215000 China; 2 Institutes for Systems Genetics, Frontiers Science Center for Disease-Related Molecular Network, West China Hospital, Sichuan University, Chengdu, 610212 China

**Keywords:** Kidney transplantation, Allograft rejection, miRNA biomarkers, Multi-omics network modeling, Systems biology

## Abstract

**Background:**

Kidney transplantation is an optimal method for treatment of end-stage kidney failure. However, kidney transplant rejection (KTR) is commonly observed to have negative effects on allograft function. MicroRNAs (miRNAs) are small non-coding RNAs with regulatory role in KTR genesis, the identification of miRNA biomarkers for accurate diagnosis and subtyping of KTR is therefore of clinical significance for active intervention and personalized therapy.

**Methods:**

In this study, an integrative bioinformatics model was developed based on multi-omics network characterization for miRNA biomarker discovery in KTR. Compared with existed methods, the topological importance of miRNA targets was prioritized based on cross-level miRNA-mRNA and protein–protein interaction network analyses. The biomarker potential of identified miRNAs was computationally validated and explored by receiver-operating characteristic (ROC) evaluation and integrated “miRNA-gene-pathway” pathogenic survey.

**Results:**

Three miRNAs, *i.e.,* miR-145-5p, miR-155-5p, and miR-23b-3p, were screened as putative biomarkers for KTR monitoring. Among them, miR-155-5p was a previously reported signature in KTR, whereas the remaining two were novel candidates both for KTR diagnosis and subtyping. The ROC analysis convinced the power of identified miRNAs as single and combined biomarkers for KTR prediction in kidney tissue and blood samples. Functional analyses, including the latent crosstalk among HLA-related genes, immune signaling pathways and identified miRNAs, provided new insights of these miRNAs in KTR pathogenesis.

**Conclusions:**

A network-based bioinformatics approach was proposed and applied to identify candidate miRNA biomarkers for KTR study. Biological and clinical validations are further needed for translational applications of the findings.

**Supplementary Information:**

The online version contains supplementary material available at 10.1186/s12967-021-03025-8.

## Background

Kidney transplantation is the most effective procedure for clinical treatment of patients with end-stage kidney failure. Based on the stable work of transplanted kidney, electrolyte and acid–base balance could be maintained and patients no longer need the support of dialysis to eliminate the metabolic waste. However, due to the immune response of human body, kidney transplant rejection (KTR), including antibody-medicated rejection (AMR) and T cell-mediated rejection (CMR), is observed to have irreversible effects or even results in the loss of renal allograft function [1]. Although a series of clinical symptoms, e.g., increased serum creatinine, decreased urine output, distending pain in the transplantation area, and fever, are measured for indicating KTR occurrence, the specificity is still limited since the infection and drug response could cause similar phenotypes. Thus, screening sensitive biomarkers to predict and monitor the development of rejection after allogeneic kidney transplantation is of great significance.

MicroRNAs (miRNAs) are small single-stranded non-coding RNAs with the potential as biomarkers for a variety of pathologies. Recent studies found that miRNAs are functional regulators and indicators in the immune processes related to kidney transplantation. For example, Liu et al. reported that downregulated miR-10b could mediate rejection of renal allografts through inhibiting BCL2L11 expression [2]. Jin et al. decoded a novel mechanism between miR-650 and BCL11B for preventing rejection after kidney transplantation [3]. Based on the meta-analysis and F344-Lewis rat kidney transplantation model, Liang et al. explored the diagnostic role and dynamic change of miR-155 in acute rejection [4]. Although the experimental evidences are accumulated, few of these studies focused on the multi-regulatory patterns between miRNAs and mRNAs, and miRNA-mRNA associations are identified without thoroughly weighting miRNA interactions as structural knowledge for biomarker prioritization [5].

It is widely acknowledged that the interplay between miRNAs and mRNAs forms large-scale miRNA-mRNA regulatory networks and dynamic changes in such network systems provide functional insights in disease prediction [6]. In the era of big biomedical data and artificial intelligence, computer-aided biomarker discovery has become a new frontier to model and decipher complex miRNA-mRNA regulations [7, 8]. Many network topology-based theories are proposed and characterized to extract key miRNA signatures from the noisy background for translational researches. For example, miRNAs with more targets in the network are often found to be important in regulation, and the Hub property is commonly selected for biomarker identification [9]. Compared with this approach, in our previous work miRNAs with significantly strong single-line regulatory power were measured for their possibility as biomarkers [10]. These findings increase the understanding in miRNA biology, however, most of them focus solely on cancer applications, and the framework needs to be expanded by integrating feature parameters from multi-omics network systems such as the downstream protein–protein interaction (PPI) network (PPIN) for cross-level functional survey.

**Fig. 1:**
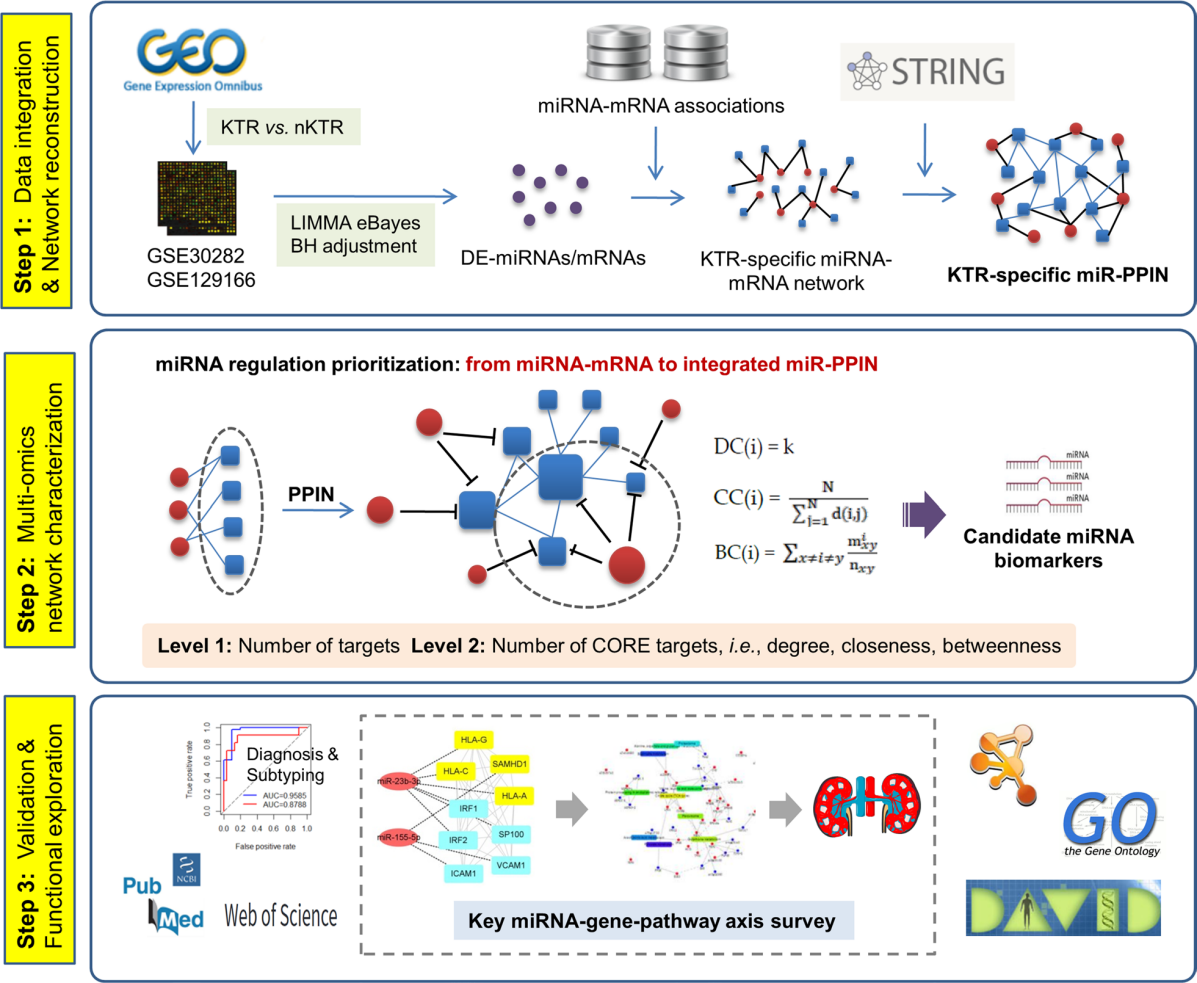
The schematic workflow of this study

In this study, we proposed a novel bioinformatics approach by characterizing and integrating topological features from both miRNA-mRNA and PPI networks to screen miRNA biomarkers and mechanisms for predicting rejection after allogeneic kidney transplantation. As shown in Fig. 1, in the method a KTR-specific miRNA-mRNA network was first developed to evaluate the binary regulatory modes between miRNAs and mRNAs. Then KTR-specific mRNAs were extracted to investigate their interactions based on PPI data, and a cross-level miRNA-mRNA-PPI network (miR-PPIN) was finally constructed to identify miRNAs and infer key miRNA-mRNA regulations in KTR. Compared with traditional approaches equally treating the contribution of different miRNA targets in the network for model training [11], in this pipeline the structural importance of target genes was mainly prioritized based on their topological activities in PPIN, and the targets in the centre of PPIN, i.e., high degree, closeness, and betweenness, were classified as CORE factors for miRNA regulatory power measurement.

## Materials and methods

### Data collection and processing

The miRNA and mRNA datasets were collected and downloaded from Gene Expression Omnibus (GEO) [12]. As illustrated in Table 1, GSE30282 contains a total of 65 post-transplant biopsy samples profiled on Affymetrix Multi-species miRNA-1 Array. In this study AMR, CMR and the normal control samples were selected [13]. In GSE129166, peripheral blood samples and kidney allograft biopsies were chosen for genome-wide gene expression analysis using Affymetrix Human Genome U133 Plus 2.0 Array [14]. To ensure the consistency of sample sources for model training, kidney allograft biopsy samples with clear pathologic characteristics, i.e., AMR or CMR (borderline samples were removed), were analyzed. In addition, GSE115816 with 6 AMR, 4 CMR and 6 stable graft function samples screened by Illumina HiSeq 2500 was used as an independent validation dataset to evaluate and compare the biomarker power of identified miRNAs for KTR prediction and subtyping in blood samples.

**Table 1:** Datasets used in this study

Category	Type	GEO accession	Sample source	Platform	Sample number
Prediction	miRNA	GSE30282	kidney allograft biopsy	GPL8786	AMR: 11; CMR: 30; Normal: 10
Prediction	mRNA	GSE129166	kidney allograft biopsy	GPL570	KTR: 24; Normal: 60
Validation	miRNA	GSE115816	Whole blood	GPL16791	AMR: 6; CMR: 4; Normal: 6

The differentially expression analysis were performed on the normalized data to extract differentially expressed (DE) miRNAs and mRNAs between KTR and normal groups. According to the comparison of methods for microarray-based DE-gene identification [15], the empirical bayes (eBayes) method was recommended for raw p-value calculation [16] and the Benjamini–Hochberg false discovery rate (FDR) was calculated for multiple testing and p-value adjustment. For the gene related to multiple probes, the probe with the most significant variation was assigned. The cutoff for DE-miRNA and DE-mRNA extraction was set as the adjusted p-value (adj.p-value) < 0.05.

The miRNA-mRNA regulatory data were retrieved from our previous work, where a human global miRNA-mRNA network was constructed based on the integration of miRNA-mRNA pairs from both experimentally validated and computationally predicted databases [10]. For human PPI data, the online tool STRING (v11.0) was applied and PPIs with the score ≥ 0.7 (high confidence) and active interaction sources except text-mining were chosen for network modeling and analysis [17].

### Multi-omics network characterization and miRNA biomarker identification

As described in Fig. 1, an integrated KTR-specific network was constructed by merging interactive data at two omics levels to quantify the power of miRNAs for gene regulation:

First, the DE-miRNAs and DE-mRNAs were mapped onto the human global miRNA-mRNA network, and a KTR-specific miRNA-mRNA network was extracted to measure miRNA regulation at the post-transcriptional level.

Second, it should be acknowledged that genes in the network could influence the downstream biological processes and eventually cause disease phenotypes via interacting with each other. To better prioritize the interactive activity of genes regulated by miRNAs, a KTR-specific PPI network was further developed by integrating KTR-specific mRNAs with human PPI data using STRING online [17]. Here three topological features, i.e., degree centrality (DC), closeness centrality (CC), and betweenness centrality (BC), were calculated to indicate the structural significance of genes in PPIN [18]:

$${\text{DC}}\left( {\text{i}} \right) \, = {\text{ k}}$$

where k is the number of edges that are linked to a given node i. Nodes with high DC are critical in the network.

$${\text{CC}}\left( {\text{i}} \right) \, = \frac{{\text{N}}}{{\sum\nolimits_{{\text{j = 1}}}^{{\text{N}}} {{\text{d}}\left( {{\text{i}},{\text{j}}} \right)} }}$$

where N is the total number of nodes in the network, and d(i, j) represents the distance between i and j. This index represents how close the given node is to all the other nodes in the network. In general, the node with high CC holds the optimum position for evaluating the information flow.

$${\text{BC}}\left( {\text{i}} \right) \, = \sum\nolimits_{x \ne i \ne y} {\frac{{{\text{m}}_{xy}^{i} }}{{{\text{n}}_{xy} }}}$$

where n_xy_ is the number of the shortest paths connecting node x and y, and m^i^_xy_ is the number of the shortest paths connecting node x and y that contain the given node i. Since the interaction of two non-adjacent nodes can be influenced by nodes that lie between them, this index indicates the significance of a given node based on the number of penetrated shortest paths.

In this study, genes whose DC, CC and BC are greater than their average values are defined as CORE set in the network (see Additional file 1), and the KTR-specific miRNA-mRNA network were upgraded by merging PPIN to a cross-level miRNA-mRNA-PPI network (miR-PPIN). In this novel framework, the structural importance of target genes is prioritized according to their contributions in the network, and miRNAs with more CORE targets have the theoretical priority for biomarker discovery.

Based on the above definitions, two feature parameters, i.e., Number of ALL Targeted Genes (NTG_A_) and Number of Targeted CORE Genes (NTG_C_), were respectively defined, where the former is numerically equal to the number of genes regulated by miRNAs and the latter is the number of CORE genes targeted by a given miRNA. As hub genes are reported to be functionally important in the network system [19], miRNAs with high NTG_A_ values have strong power in regulation. Moreover, NTG_C_ index strengthens such regulatory pattern by reasonably weighting the contribution of target genes across multi-omics networks. Hence candidate miRNA biomarkers were computationally identified with the following two steps:

Step 1: miRNAs with significantly high NTG_A_ values (p-value < 0.05, Wilcoxon signed-rank test) were extracted from the KTR-specific network.

Step 2: miRNAs with significantly high NTG_C_ values (p-value < 0.05, Wilcoxon signed-rank test) were selected from those screened in Step 1 as candidate biomarkers for expression and functional validation.

### ROC analysis and performance validation

The receiver-operating characteristic (ROC) analysis was performed and compared using the statistical tool MedCalc (v20.009) at two levels, i.e., the identified miRNAs as single predictors and a combined signature that incorporated all identified miRNAs. Here the Logistic regression was applied to predict the value of combined signature based on the expression data of each miRNA variables. The area under ROC curve (AUC) was calculated to quantify the predictive power of biomarkers for KTR diagnosis (KTR vs. nKTR) and subtyping (AMR vs. CMR).

The potential of miRNAs as biomarkers was further validated by literature-based pathogenic survey, and keywords including “kidney/renal transplantation”, “rejection”, “immune response”, etc. were used to investigate the associations of identified miRNAs with KTR through text-mining in PubMed and Web of Science.

### Knowledge-guided key regulatory module extraction and functional exploration

The pathogenesis of identified miRNAs was explored based on a three dimensional “miRNA-gene-pathway” paradigm. First, functional targets of biomarker miRNAs were retrieved from KTR-specific miR-PPIN network for Gene Ontology (GO) enrichment at the biological process (BP) level using the Database for Annotation, Visualization and Integrated Discovery (DAVID, v6.8) online [20]. The criterion for significant term selection was set as p-value < 0.05. Then PPIs in KTR-specific miR-PPIN were clustered by the plug-in Molecular Complex Detection (MCODE) of Cytoscape for molecular complex analysis with default thresholds [21]. Finally, significant clusters were identified and key regulatory modules were extracted by integrating BP knowledge and miRNA regulations for translational “genotype-phenotype” etiologic understanding.

## Results

### Global features of KTR-specific miR-PPIN

**Fig. 2:**
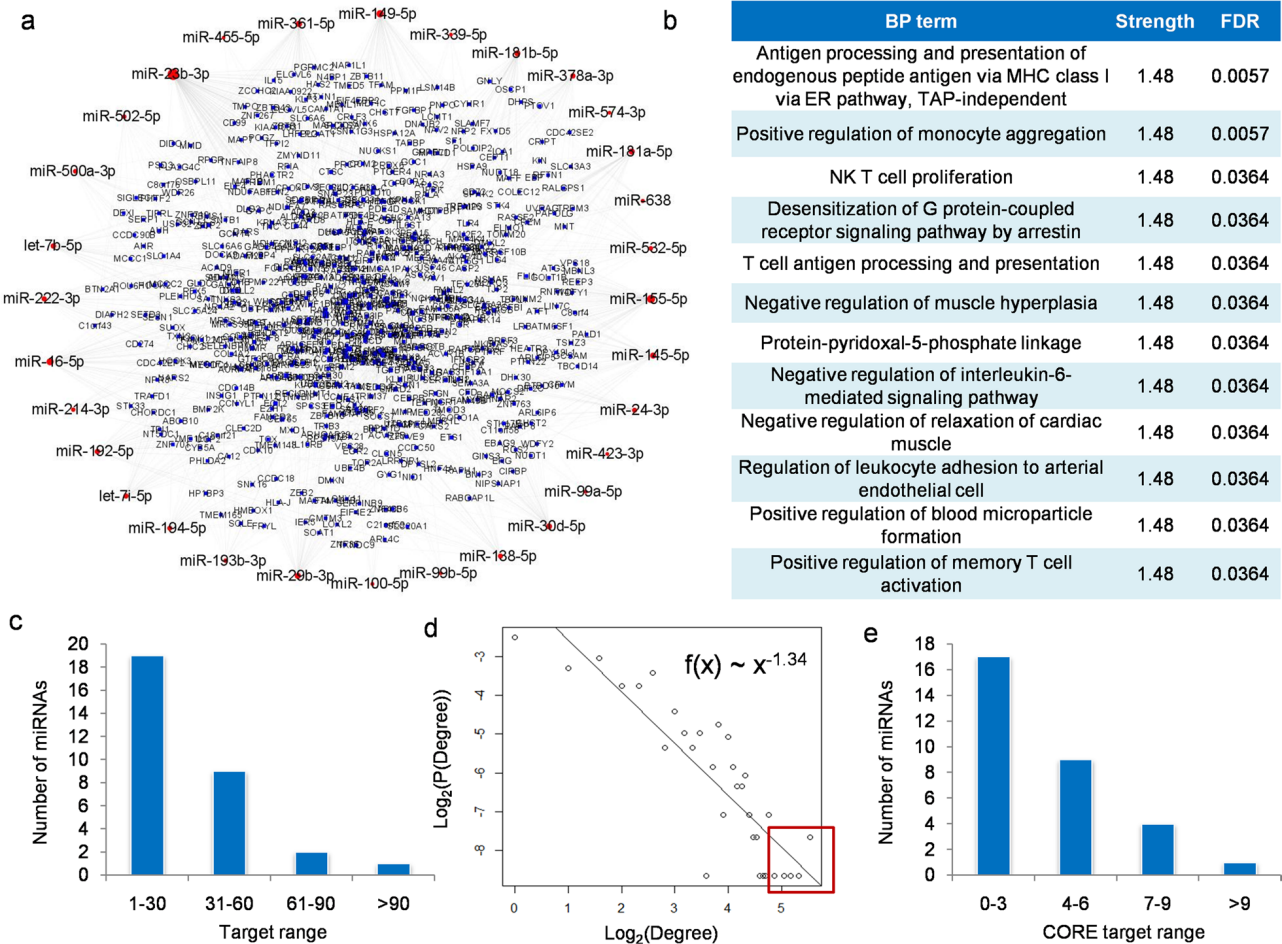
Structural characterization and prioritization of KTR-specific miR-PPIN: (**a)** Overview of the network. The red and blue nodes represent miRNAs and genes, respectively; **(b)** BP terms significantly enriched by miR-PPIN with the highest strength according to STRING online; **(c)** Distribution of targets at miRNA-mRNA level. miRNAs with more targets were fewer in number; **(d)** Degree distribution of genes at PPI level, where P (Degree) is the fraction of genes in the network with the given Degree to others. Both Degree and P (Degree) were 2-based log transformed; **(e)** Distribution of CORE targets at miR-PPIN level. miRNAs with more CORE targets were fewer in number

In this study, a KTR-specific miR-PPIN network was constructed from the integration of DE-miRNA/mRNA, miRNA-mRNA and PPI data for systems-level characterization of miRNA-mediated interactions in KTR (see Additional file 2). As shown in Fig. 2a, the network contains a total of 949 miRNA-mRNA pairs and 1,447 PPIs among 31 miRNAs and 642 genes that were abnormally expressed in KTR. Based on STRING analysis [17] as described in Fig. 2b, the genes in the network were significantly enriched in T cell-related BP terms such as NK T cell proliferation, T cell antigen processing and presentation, positive regulation of memory T cell activation, et al., which indicated the specificity of identified network in KTR development [22, 23].

To characterize the global features of miRNAs and genes in the network, the degree distribution was analyzed at miRNA-mRNA and PPI level, respectively. As illustrated in Fig. 2c and d, miRNAs with more targets in miRNA-mRNA network were fewer, and most of the targets tended to locate at border sites in PPIN as the degree of nodes followed the power-law distribution with the slope of -1.34. Based on these findings, the structural importance of target genes was then prioritized according to their degree, closeness and betweenness properties in PPIN, and the highly prioritized targets were classified as CORE gene set to strengthen miRNA regulation (see Materials and methods). Finally, features in these two network systems were collaboratively measured, and the number of CORE targets by miRNAs was calculated in miR-PPIN. As shown in Fig. 2e, there were very few miRNAs that regulated more CORE genes and it could be a potential clue for biomarker discovery.

### Biomarker miRNAs for KTR diagnosis and subtyping

**Fig. 3:**
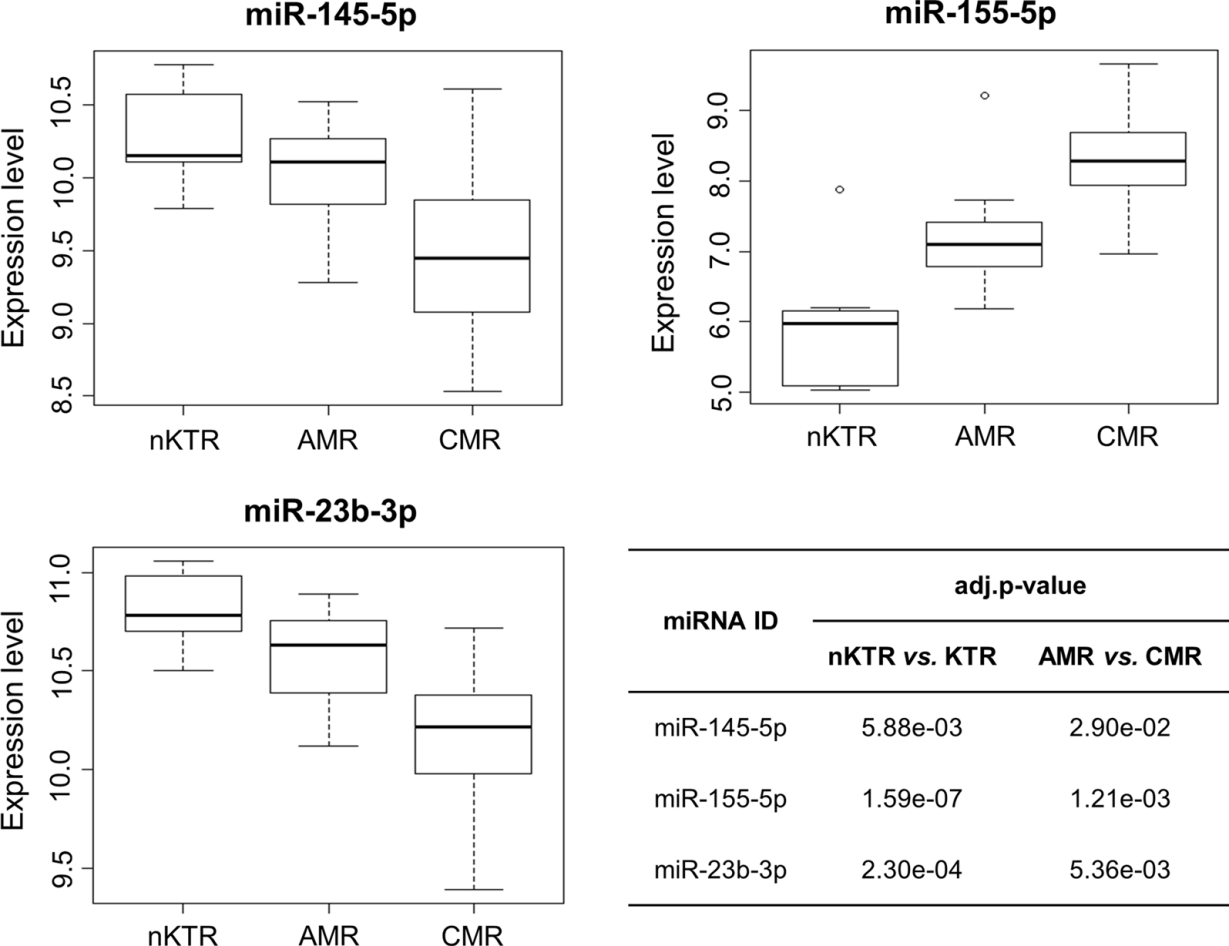
The expression pattern of identified miRNA biomarkers in nKTR (normal control), AMR and CMR group. The expression data were 2-based log transformed from the selected prediction dataset (GSE30282)

In this study, a total of three miRNAs, i.e., miR-145-5p, miR-155-5p, and miR-23b-3p, were identified as candidate biomarkers for KTR prediction based on multi-omics network characterization with the newly defined topological parameters and screening criteria, i.e., significantly high NTG_A_ and NTG_C_. As illustrated in Table 2 and Fig. 3 respectively, miR-145-5p and miR-23b-3p were downregulated in KTR group, whereas miR-155-5p was overexpressed in KTR especially in CMR.

**Table 2:** The identified miRNAs as candidate biomarkers

miRNA ID	Expression	NTG_A_ (p-value)	NTG_c_ (p-value)
miR-145-5p	Down	44 (6.75e-04)	9 (7.81e-03)
miR-155-5p	Up	77 (1.84e-07)	10 (1.95e-03)
miR-23b-3p	Down	131 (9.31e-10)	9 (7.81e-03)

**Fig. 4:**
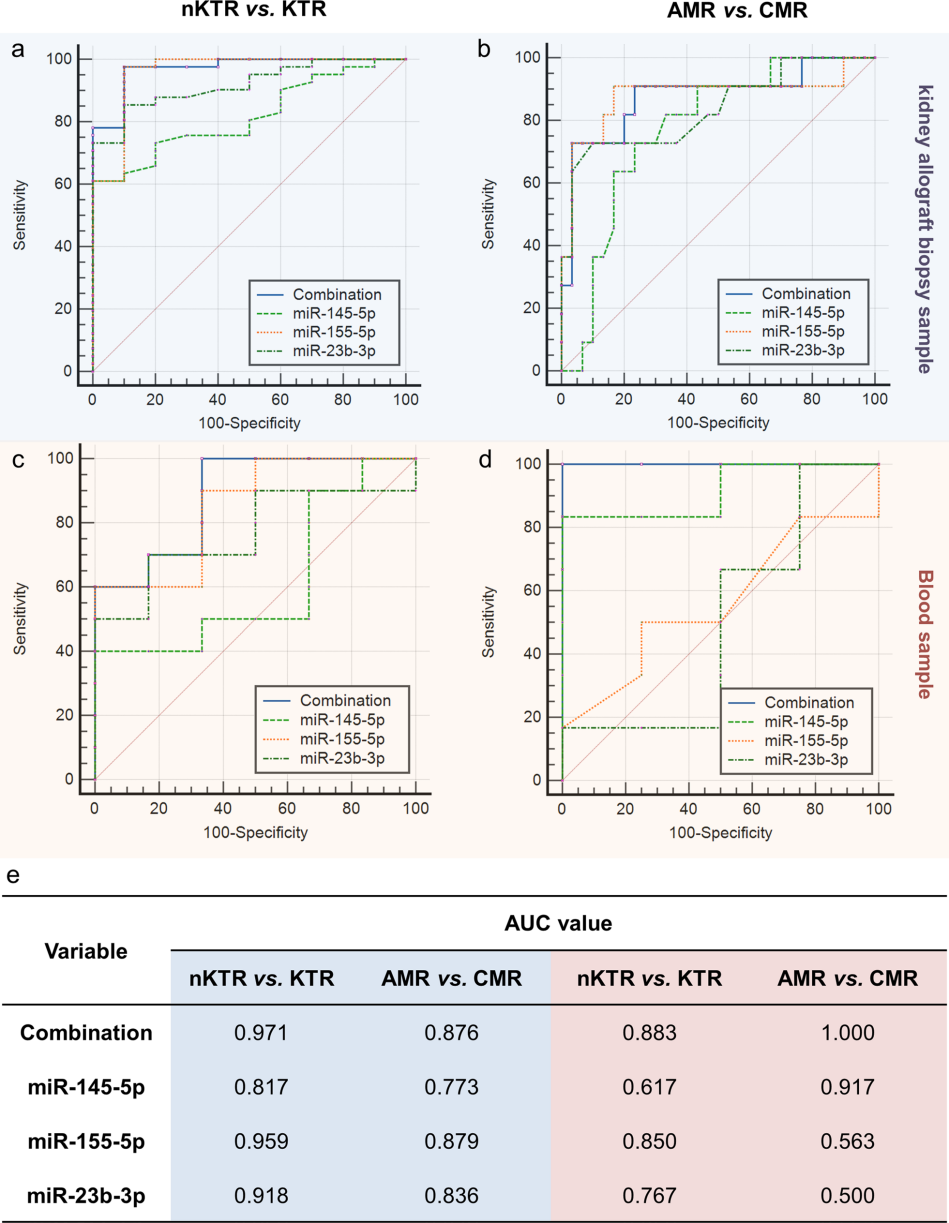
The ROC analysis of identified miRNA biomarkers and their combined signature for KTR diagnosis and subtyping: **(a)** and **(b)** The performance in kidney allograft biopsy samples (GSE30282); **(c)** and **(d)** The performance in blood samples (GSE115816); **(e)** Comparison of AUC values among different conditional groups. Blue and red shadings represent biopsy and blood sample respectively

The ROC analyses were performed both on the training and an independent validation dataset to evaluate the potential of identified miRNAs and the miRNA combination for KTR diagnosis and subtyping. At single miRNA level, as drawn in Fig. 4a, b and e, all the three miRNAs showed strong power (AUC > 0.8, average AUC = 0.898) in differentiating kidney allograft biopsy samples of KTR and non-KTR (nKTR, normal control), and they had comparable ability for KTR subtyping, i.e., AMR and CMR (AUC > 0.75, average AUC = 0.829). In the validation set, the miRNAs were measured using blood samples since blood can be obtained noninvasively compared with kidney tissues. As shown in Fig. 4c, d and e, miR-155-5p and miR-23b-3p tended to outperform on KTR prediction (AUC > 0.75), and miR-145-5p reached the AUC of 0.917 for AMR and CMR subtyping. Compared with single biomarkers, the combination of three miRNAs improved the overall performance in different groups, which indicated the potential of the three miRNAs as a combined signature for KTR management.

It should be noticed that the AUC values of identified miRNAs were highly heterogeneous between tissue and blood groups. In addition to the differences in sample source, the number and size of dataset is another important consideration. Therefore large-sample-based consistency analysis should be conducted for further functional validation.

### Literature-based functional validation and comparison

The literature-based survey was conducted to validate the performance of the bioinformatics model and investigate the function of identified miRNAs. Among them, miR-155-5p is a well-studied star related to dysfunctional allograft status in kidney transplantation. For example, Anglicheau et al. demonstrated that the intragraft expression of miRNAs was strongly associated with mRNAs and the disorder in such regulation led to acute rejection and affected kidney allograft function. They found that miR-155-5p (namely miR-155) was overexpressed in KTR biopsy samples, which could serve as a diagnostic biomarker for indication of kidney allograft status [24]. Similar to this result, the overexpression of miR-155-5p was detected in T-cell mediated rejection from a study by Soltaninejad et al., and the diagnostic role of this miRNA was suggested for CMR prediction [25]. To validate the dynamic change of miR-155-5p expression in KTR, Liang et al. established a rat kidney transplantation model and the result showed that the increased level of miR-155-5p in plasma was highly consistent with the degree of rejection development, thus this miRNA could be a useful biomarker for monitoring the functional status of allograft after kidney transplantation [4]. In addition to tissue and blood samples, the upregulation of miR-155-5p was also captured in urine from patients who experienced rejection. Based on qRT-PCR validation, the level of urinary miR-155-5p was significantly increased in patients before and during acute rejection, and this miRNA held the power to be a biomarker for early diagnosis of rejection after kidney transplantation [26].

The remaining two miRNAs, i.e., miR-145-5p and miR-23b-3p, were also reported to be functional in KTR progression. According to the RT-PCR analysis by Matz et al., the abnormal expression of miR-145-5p distinguished rejection from the normal controls and it was downregulated in patient cohorts with interstitial fibrosis/tubular atrophy (IFTA), which indicated its diagnostic role as an IFTA-specific biomarker in blood for fibrosis-caused etiology study [27]. Meanwhile, this miRNA, as well as miR-23b-3p, could distinguish acute rejection and acute pyelonephritis, which provided underlying clues for rejection identification from inflammatory conditions [28].

To summarize, miR-155-5p is a reported biomarker for KTR diagnosis and the remaining candidates also have close associations with KTR especially with kidney acute rejection processes, supporting the overall predictive performance of the proposed model. Compared with previous approaches and findings, the expression pattern of identified miRNAs in KTR group was highly consistent. More importantly, in this study a network-based computational strategy was applied to identify miRNAs with biomarker potential both in KTR diagnosis (KTR vs. nKTR) and subtyping (AMR vs. CMR), which provided novel insights in miRNA-medicated KTR pathogenesis.

### Key regulatory modules and mechanisms in KTR pathogenesis

It is widely known that immune response is a leading factor affecting the function of allograft after kidney transplantation [29, 30]. In this study immune-related BP terms, e.g., interferon-gamma-mediated signaling pathway, type I interferon signaling pathway, immune response, antigen processing and presentation, etc. were significantly enriched by biomarker miRNA targets including HLA-A, HLA-C, HLA-G, SAMHD1, ICAM1, IRF1/2, etc. in KTR-specific miR-PPIN (see Additional file 3). To explore genetic interactions associated with identified BP terms, the plug-in MCODE of Cytoscape was applied to cluster functional modules for pathogenetic understanding. As illustrated in Additional file 4, a total of 18 clusters were extracted from KTR-specific PPIN based on MCODE analysis and the Cluster 2 with identified biomarker targets was selected for etiologic study.

**Fig. 5:**
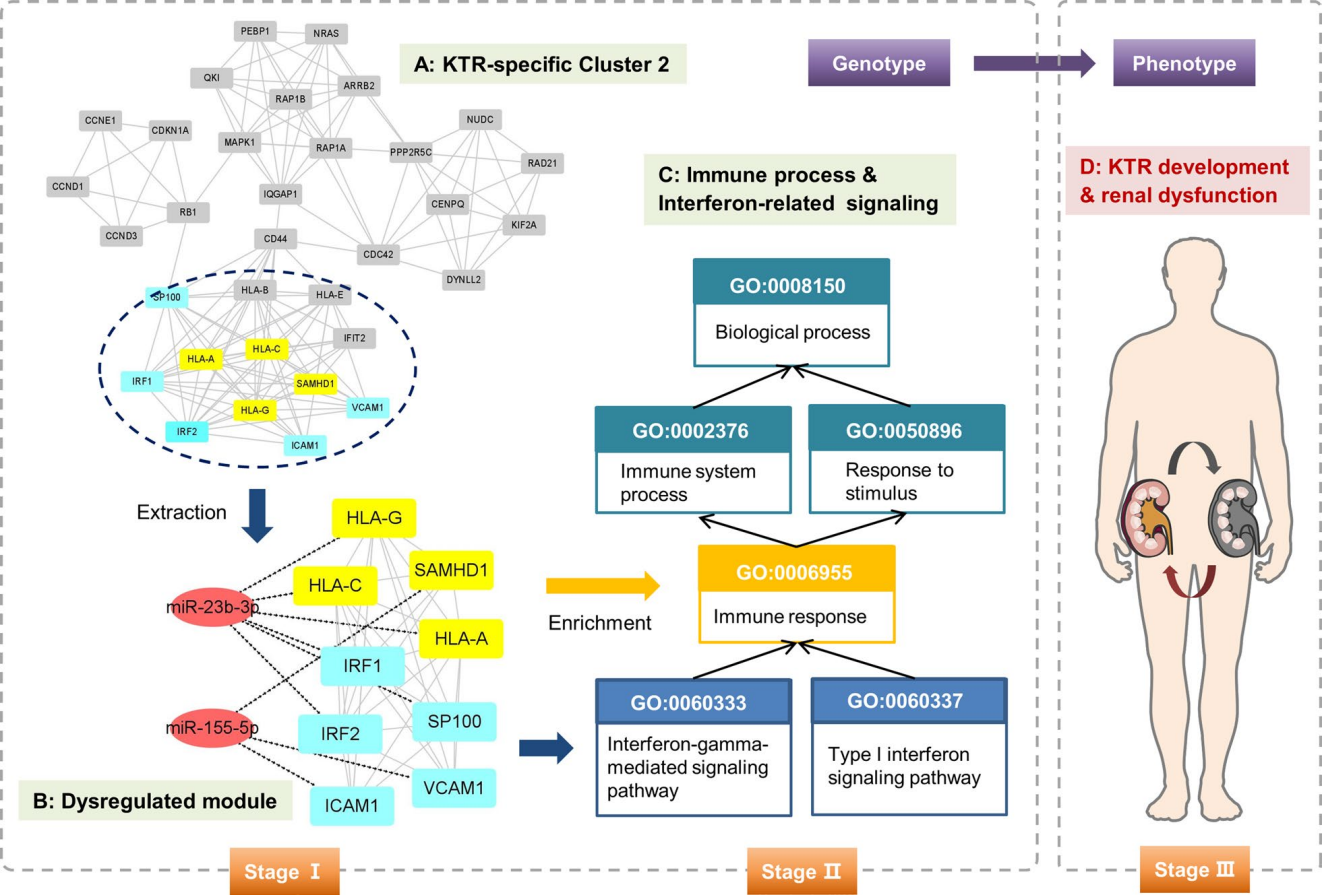
The key miRNA-mRNA regulatory module and putative mechanism associated with KTR development

As shown in Fig. 5, in KTR-specific Cluster 2 the identified targets were interacted and formed as a key module in regulating immune and interferon-related pathways. Herein HLA-A, HLA-C, HLA-G, IRF1/2 and SP100 were regulated by miR-23b-3p and the remaining three were targets of miR-155-5p, respectively. Among them, HLA (human leukocyte antigen) is known to be the expression product of human major histocompatibility complex (MHC). The mismatch of HLA is a risk factor to affect kidney allograft function and to increase the chance of rejection [31–33]. For example, Ko et al. investigated the clinical outcome of kidney transplantation with ABO and HLA incompatibilities. The result found that ABO and HLA incompatibilities could increase the probability of infection-medicated rejection and influence the overall survival both for patient and graft [31]. Moreover, HLA donor-specific antibodies could induce inflammation, vessel injury and AMR by binding to vascular endothelial cells of the allograft [34]. As three important members in HLA class I heavy chain paralogues, HLA-A, HLA-C and HLA-G were found to be potentially regulated by miR-23b-3p in this study. Here HLA class I molecules have central functions in the immune system, and they can be detected by cytotoxic T cells via presenting peptides originated from the endoplasmic reticulum lumen. In particular, Janssen et al. found that polymorphisms in donor derived HLA-G had significant effects on acute rejection after kidney transplantation, and 14-bp ins/ins and the + 3142GG genotypes may be protective factors to decrease KTR [35]. Hence the signaling of miR-23b-3p/HLA/immune axis offers novel insights in KTR genesis. In addition to HLA groups, ICAM1 and VCAM1 are well investigated in the function of transplanted kidney. For example, the immunological response after kidney transplantation would increase the endothelial expression of ICAM1 and VCAM1, and the rs5498 ICAM1 polymorphism and the C allele of the rs1041163 in VCAM1 highlighted the rejection risk and long-term allograft failure [36, 37]. Moreover, as an inflammatory protein, ICAM1 plays active roles in kidney chronic rejection, and the ICAM1-based network biomarker accelerated the precision classification of different KTR phenotypes [38]. Last but not least, IRF1 is a transcription factor and it mediated MHC induction and regulated resistance to necrosis in KTR, which would be crucial for the interventions against rejection [39].

Based on these findings, the putative “miRNA-gene-pathway” axis was inferred at the computational level and more insightful mechanisms in KTR pathogenesis need to be experimentally validated in the future work.

## Discussion

Allograft rejection is a common complication after kidney transplantation. The early detection and precise subtyping of rejection signatures are of clinical significance for personalized therapy. As a class of non-coding RNAs with regulatory role in multiple biological processes, miRNAs are well reported to be potential biomarkers for predicting rejection response in kidney transplantation [40, 41]. However, most of the studies are experimental, which lacks deep insights in the global interactions between miRNAs as well as target genes for systems-level pathogenesis understanding.

In this study, a novel bioinformatics framework was proposed based on integration of microarray data and multi-omics network knowledge for cross-level deciphering of RNA interplay during KTR development. In methodology, a KTR-specific miR-PPIN was constructed and topological characterization was conducted to uncover theoretical hypothesis for miRNA biomarker discovery. Compared with traditional methods that equally rank the contribution of miRNAs in miRNA-mRNA network [42, 43], the network system in this study was expanded by reasonably weighting the interaction among targets of miRNAs based on downstream PPI analysis. Hence in this model, the topological importance of genes targeted by miRNAs was prioritized according to their structural characteristics in PPIN. Two feature parameters, i.e., NTG_A_ and NTG_C_, were defined for biomarker identification. Finally, a total of three miRNAs, i.e., miR-145-5p, miR-155-5p, and miR-23b-3p, were screened as candidate biomarkers for monitoring the rejection after kidney transplantation.

Among the three candidates, miR-155-5p was significantly overexpressed in rejection groups, whereas the remaining two were downregulated in KTR process. The ROC analysis showed that all the three miRNAs distinguished KTR from the normal controls with the AUC > 0.8 in kidney allograft biopsy samples, and they achieved comparable performance on AMR and CMR subtyping (AUC > 0.75) as well. Meanwhile, miR-155-5p/miR-23b-3p (AUC > 0.75) and miR-145-5p (AUC > 0.9) were respectively found to be powerful for KTR prediction and subtyping in blood, indicating the noninvasive diagnostic potential of identified miRNAs. In addition to single biomarkers, the three miRNAs could serve as a combined signature to improve the overall performance for KTR management.

According to literature reports, miR-155-5p had found to be a diagnostic biomarker and a prognostic factor for predicting allograft status and rejection [4, 26, 44]. The remaining miRNAs were also found to be dysfunctional during KTR development. Compared with existing findings, in this study the miRNAs showed the predictive power both in KTR diagnosis and subtyping. Moreover, miR-23b-3p and miR-155-5p were inferred to regulate immune and interferon-medicated pathways via targeting the module of HLA-related and ICAM1/VCAM1 genes respectively, which highlighted the novel putative mechanisms for KTR understanding.

The limitations in this study should be carefully considered although meaningful results were obtained and analyzed. First, the importance and contribution of miRNAs and genes in the network were prioritized based on their structural characteristics, the functional significance of genes in immune processes related to rejection responses needs to be investigated and quantified to improve the sensitivity of miRNAs in KTR management. Second, in addition to KTR and the normal controls, samples from other conditional phenotypes such as infection-mediated or drug toxicity-induced delated graft function (DGF) should be included and compared to test the biomarker specificity of identified miRNAs in rejection since similar clinical symptoms, e.g., decreased amount of urine and increased level of serum creatinine, often occurred in the early stage both of KTR and DGF [45, 46]. Finally, it is potentially important that the biomarkers originally identified from tissue biopsies were able to be validated in blood samples as blood could be easily obtained for clinical translation. However, the ROC result from tissue and blood group tended to be heterogeneous in this study. Although many studies reported that the concordance rate for detection of gene variant and expression may be affected by the type and source of sample data [47–49], it should be admitted that the blood sample set used for comparison was relatively small. Hence the biomarker significance of identified miRNAs in blood needs to be further explored with the accumulation of enough public data to decrease the risk of model overfitting. On the other hand, large-sample-based multi-center experimental validation and pathogenetic survey are expected to be performed for future translational applications.

## Conclusion

In this study, a total of three miRNAs, i.e., miR-145-5p, miR-155-5p, and miR-23b-3p, were screened as candidate biomarkers for diagnosis and subtyping of allograft rejection after kidney transplantation based on an integrated bioinformatics model with multi-omics network characterization. The potential regulatory mechanisms of HLA-related genes, immune signaling pathways and identified miRNAs were computationally discovered for pathogenic understanding. In the future work, molecular experiments using human samples will be performed for further clinical validation.

## Supplementary Information

**Additional file 1. **CORE genes identified in this study.

**Additional file 2. **The constructed KTR-specific miR-PPIN.

**Additional file 3.** Significantly enriched BP terms by identified miRNA biomarkers.

**Additional file 4. **Clusters extracted from KTR-specific PPIN using MCODE.

## Data Availability

The data generated or analyzed during this study are available from the corresponding authors upon reasonable request.

## Abbreviations

KTR: Kidney transplant rejection
miRNA: MicroRNA
AMR: Antibody-medicated rejection
CMR: Cell-mediated rejection
PPIN: Protein–protein interaction network
miR-PPIN: MicroRNA-mRNA-PPI network
GEO: Gene Expression Omnibus
DE: Differentially expressed
eBayes: Empirical bayes
FDR: False discovery rate
adj.p-value: Adjusted p-value
DC: Degree centrality
CC: Closeness centrality
BC: Betweenness centrality
NTG_A_: Number of ALL Targeted Genes
NTG_C_: Number of Targeted CORE Genes
ROC: Receiver-operating characteristic
AUC: Area under ROC curve
GO: Gene Ontology
BP: Biological process
DAVID: The Database for Annotation, Visualization and Integrated Discovery
MCODE: Molecular Complex Detection
IFTA: Interstitial fibrosis/tubular atrophy
HLA: Human leukocyte antigen
MHC: Major histocompatibility complex
DGF: Delated graft function
